# PBX1-SIRT1 Positive Feedback Loop Attenuates ROS-Mediated HF-MSC Senescence and Apoptosis

**DOI:** 10.1007/s12015-022-10425-w

**Published:** 2022-08-12

**Authors:** Yuan Wang, Yutong Sui, Ye Niu, Dan Liu, Qi Xu, Feilin Liu, Kuiyang Zuo, Mingsheng Liu, Wei Sun, Ziyu Wang, Zinan Liu, Fei Zou, Jiahong Shi, Xiaomei Liu, Jinyu Liu

**Affiliations:** 1 Department of Toxicology, School of Public Health, Jilin University, 1163 Xinmin Avenue, Changchun, 130021 Jilin China; 2 Eye Center, The Second Hospital of Jilin University, Changchun, 130021 Jilin China; 3 Department of Neuroscience, Mayo Clinic, Jacksonville, FL 32224 USA; 4 Department of Ultrasound, The China-Japan Union Hospital of Jilin University, Changchun, 130021 Jilin China

**Keywords:** Hair follicle mesenchymal stem cells, PBX 1, SIRT1, PARP1, DNA damage, Senescence, Apoptosis

## Abstract

Stem cell senescence and depletion are major causes of aging and aging-related diseases. The NAD (Nicotinamide adenine dinucleotide) – SIRT1 (Silent Information Regulator 1) – PARP1 (Poly (ADP-ribose) polymerase-1) axis has gained interest owing to its significant role in regulating stem cell senescence and organismal aging. A recent study from our lab showed that pre-B-cell leukemia transcription factor1 (PBX1) overexpression attenuates hair follicle-derived mesenchymal stem cells (HF-MSCs) senescence and apoptosis by regulating ROS-mediated DNA damage via PARP1 downregulation; thus, suggesting that PARP1 downregulation is a common manifestation of the roles of both PBX1 and SIRT1 in HF-MSCs senescence attenuation, and implying a potential link between PBX1 and SIRT1. To this end, HF-MSCs overexpressing PBX1, overexpressing both PBX1 and PARP1, downregulating SIRT1, and overexpressing PBX1 as well as downregulating SIRT1 were generated, and senescence, apoptosis, DNA damage, and repair biomarkers were analyzed. Our results showed that (1) PBX1 overexpression alleviated HF-MSCs senescence and apoptosis accompanied by SIRT1 upregulation, PARP1 downregulation, and increased intracellular NAD and ATP levels. (2) SIRT1 knockdown enhanced cellular senescence and apoptosis, accompanied by increased ROS accumulation, DNA damage aggravation, and decreased intracellular NAD and ATP levels. (3) PBX1 overexpression rescued HF-MSCs senescence and apoptosis induced by SIRT1 knockdown. (4) PBX1 rescued PARP1 overexpression-mediated ATP and NAD depletion, accompanied by increased SIRT1 expression. Collectively, our results revealed that a positive interaction feedback loop exists between PBX1 and SIRT1. To the best of our knowledge we are the first to report that there is a PBX1-SIRT1-PARP1 axis that plays a critical role in alleviating HF-MSCs senescence and apoptosis. We provide a new perspective on the mechanisms underlying stem cell senescence as well as age-related disease prevention and treatment.

**Graphical Abstract:**

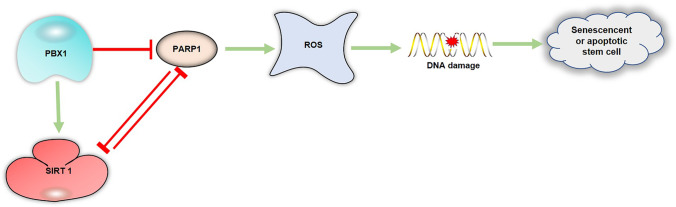

**Supplementary Information:**

The online version contains supplementary material available at 10.1007/s12015-022-10425-w.

## Introduction

Tissue homeostasis is regulated by stem cells, and age-related tissue degeneration is closely associated with a decline in stem cell function and number [1]. Hence, stem cell senescence and depletion are regarded as major risk factors for organismal aging and age-related diseases. DNA damage is a major cause of stem cell senescence. Cells maintain genomic stability via the DNA damage response (DDR) [2]. In addition to the repair mechanisms, another essential factor influencing DNA damage is the state of chromatin, which may affect the sensitivity of DNA to DNA-genotoxic agents and interfere with access to DDR signaling and DNA repair factors [2]. Histone acetylases (HATs) and deacetylases (HDACs) can identify DNA damage sites, contribute to the recruitment of DNA repair proteins, silence transcription during the repair process, and restore the chromatin state after repair [3, 4].

SIRT1 is a highly conserved NAD-dependent lysine deacetylase and ADP-ribosyl transferase. It is widely recognized as a crucial epigenetic regulator [5, 6] and participates in numerous biological processes, including gene silencing, DNA repair, metabolic regulation, cell cycle regulation, apoptosis, inflammation [7, 8], autophagy, cellular senescence [9], heterochromatin formation, and in protection against various human diseases. SIRT1 is thus widely involved in the regulation of cellular senescence and contributes to organism longevity via acetylation and deacetylation of substrates, altering their transcriptional and enzymatic activities as well as corresponding protein levels [7, 10]. Thus, SIRT1 upregulation can affect the regulation of cellular senescence in aging and aging-related diseases.

A strategy to activate SIRT1 relies on increasing the NAD levels. An increase in NAD is mediated via the repression of major cellular NAD-consuming enzymes, such as PARP-1 and CD38, or the direct supplementation of NAD precursors such as nicotinamide mononucleotide (NNM) and nicotinamide riboside (NR). Furthermore, increasing the NAD levels can help maintain telomere integrity and promote the DNA damage response in a SIRT1-dependent manner, including the activation of genes involved in DNA damage repair and direct chromatin modifications at DNA breaks through the recruitment of DNA damage repair proteins [11, 12]. Therefore, SIRT1 activation or upregulation is expected to represent an effective method for tissue homeostasis maintenance, stem cell senescence attenuation, lifespan prolongation, and aging-related disease amelioration.

PBX1 (Pre-B-cell leukemia transcription factor1) is a key transcription factor that regulates cell fate, particularly stem cell proliferation, apoptosis, senescence, and differentiation [13–15]. Our previous study showed that PBX1 overexpression significantly attenuates HF-MSCs senescence and apoptosis, accompanied by a downregulation of PARP1 expression. PARP1 can be activated by oxidative, metabolic, and genotoxic stresses and directs cells to specific fates depending on the type and intensity of stress [16]. Although PARP1 is generally regarded as a DNA damage repair enzyme, it also has equally important functions in the regulation of apoptosis or necrotic cell death, which depletes large amounts of NAD and ATP [17–19]. Considering that PARP1 is a major cellular NAD consumer, PARP1 levels can directly affect the activity of other NAD-dependent enzymes, such as SIRT1, which plays a crucial role in the protection against oxidative injury. NAD depletion mediated by PARP1 overactivation attenuates the deacetylase activity of SIRT1, which is implicated in longevity and cellular protection since NAD is a replaceable substrate of SIRT1.

Nevertheless, whether PBX1 attenuates HF-MSCs senescence and apoptosis by regulating the SIRT1-PARP1 axis remains unknown. To this end, this study aimed to develop HF-MSCs overexpressing PBX1, overexpressing both PBX1 and PARP1, downregulating SIRT1, or overexpressing PBX1 and downregulating SIRT1 as well as examining the biomarkers associated with senescence, apoptosis, DNA damage, and DNA repair in HF-MSCs.

## Materials and Methods

This work was approved by the Ethics Committee of the School of Public Health, Jilin University, China.

### Cell Culture

The isolation and identification of HF-MSCs were carried out as in our previous study [15, 20]. Isolated HF-MSCs were cultured in Dulbecco’s modified Eagle’s medium (Life Technologies, Gaithersburg, MD, USA) containing 10% fetal bovine serum (FBS; Hyclone, Logan, UT, USA), 2 ng/mL of basic fibroblast growth factor (Sino Biological Inc., China) and 100 U/mL penicillin–streptomycin (Hyclone). HEK 293 T cells were cultured in DMEM containing 10% FBS and 100 U/mL penicillin–streptomycin. Cells were cultured at 37 °C and 5% CO_2_.When the cells proliferated to an 80%–90% confluence, they were digested and subcultured under similar individual conditions.

### Generation of HF-MSCs Overexpressing PBX1, PARP1, and both PBX1 and PARP1

The overexpression of PBX1, PARP1, and PBX1 + PARP1 in HF-MSCs was achieved as previously described [15, 20, 21]. The human PBX1 coding region and PARP1coding region was cloned into the pLVX-IRES-mCherry lentiviral vector (Youbio, China). The 10 μg lentiviral vector was cotransfected with 7.5 μg pMD2.G and 2.5 μg psPAX2 (Addgene) into 293 T cells in a 100 mm cell culture plate using Lipofectamine (Invitrogen) 3,000 as transfection reagent. The viral particle were harvested at 48 and 72 h after transfection and concentrated by ultracentrifugation (Millipore, United States). HF-MSCs were transduced with lentiviral particles encoding PBX1 or PARP1 or both PBX1 and PARP1 in the presence of polybrene (Santa Cruz,United States) at final concentration of 10 μg/ml.

### SIRT1 Silencing

Small interfering RNA (siRNA) oligonucleotides were purchased from RiboBio (China) with sequences targeting SIRT1. HF-MSCs were transfected with 100 nM of the indicated siRNA or scrambled RNA (scRNA) using riboFECT CP Transfection Kit (RiboBio) according to the manufacturer’s instructions. The siRNA effects on the indicated protein levels were examined using western blotting.

### ATP Production Measurement

Approximately 1 × 10^6^ HF-MSCs were harvested in 200 μL cell lysis solution and centrifuged at 12,000 g for 5 min. The intracellular ATP production was assessed using an Enhanced ATP Assay kit (Beyotime Institute of Biotechnology, Shanghai, China), adhering to the manufacturer’s guidelines. Luminescence was measured using Cytation 3 (BioTek, Winooski, VA, USA).

### NAD/NADH Production Measurement

Approximately 1 × 10^6^ HF-MSCs were harvested in 200 μL NAD/NADH extraction solution and centrifuged at 12,000 g for 5 min. The intracellular NAD/NADH production was assessed using an NAD/NADH Assay Kit (Beyotime) according to the manufacturer's instructions and absorbance using Cytation 3 (BioTek).

### Senescence-Associated-β-Galactosidase and Apoptosis Assays

A cellular senescence staining kit (Beyotime) was used to detect senescence-associated (SA)-*β*-gal activity-positive cells according to the manufacturer's instructions. When the HF-MSCs reached a 75%–80% confluence in a 24-well plate, they were fixed for 15 min at room temperature and washed using phosphate-buffered saline (PBS). Next, the HF-MSCs were incubated in staining solution mix overnight at 37 °C. The next day, cells were washed three times with PBS and observed using an optical microscope (Leica, Germany). The number of β-gal–positive cells and total cells were counted from three randomly selected fields of view.

The Annexin V-FITC/7-AAD Apoptosis Detection Kit (Sungene, China) was used to detect apoptotic-positive cells according to the manufacturer's instructions. Approximately 1 × 10^5^ HF-MSCs were suspended in 100 μL binding buffer containing 5 μL Annexin V-FITC and incubated for 15 min in the dark at temperature between 18–22 °C. After incubation, 5 μL 7-AAD was added and co-incubated for 5 min under similar conditions. The HF-MSCs were detected using flow cytometry (BD Biosciences, San Jose, CA, USA).

### ROS Assay

For ROS probing, HF-MSCs were collected by centrifugation, washed with PBS, and incubated with DCFH-DA. HF-MSCs were then detected using flow cytometry (BD Biosciences) and analyzed using FlowJo software (Treestar, San Carlos, CA, USA).

### Western Blotting Assay

Approximately 8 × 10^5^ HF-MSCs were plated in a 100-mm cell culture dish in DMEM medium containing 10% FBS and 2 ng/mL bFGF. When HF-MSCs reached an 80% confluence, cells were harvested and lysed in 200 *μ*L RIPA buffer (Beyotime) supplemented with 1% protease inhibitor cocktail (CoWin Biosciences, China) and 1% phosphatase inhibitor cocktail (CoWin Biosciences) at 4 ℃ for 40 min and centrifuged at 13,000 g for 20 min at 4 ℃. The supernatant was collected and protein concentration was analyzed using an Enhanced BCA Protein Assay Kit (Beyotime). A total of 25 μg of protein per sample were loaded into each well and separated on a 10% SDS polyacrylamide gel through electrophoresis and then transferred onto polyvinylidene difluoride membranes (Millipore, Billerica, MA, USA). The membranes were incubated in 5% nonfat milk (Anchor, New Zealand) at room temperature for 45 min. The membranes were subsequently incubated with primary antibodies: PBX1 rabbit mAb (1:1000, Cell Signaling Technologies [CST], Beverly, MA, USA), P16 rabbit mAb (1:1000; ProteinTech Group, Chicago, IL USA), P21Waf1/Cip1 (12D1) rabbit mAb (1:1000; CST), P53 mouse mAb (1:1000; Santa Cruz Biotechnology, Santa Cruz, CA, USA), PARP1 rabbit mAb (1:1000; CST), AIF rabbit mAb (1:1000, CST), Sirtuin 1 mouse mAb (1:1000, CST), Cleaved Caspase 3 rabbit mAb (1:1000, CST), γH2AX rabbit mAb (1:1000, CST), GAPDH mouse mAb (1:10,000; ProteinTech Group), HRP-conjugated AffiniPure Goat Anti-Rabbit IgG (H + L) (1:5000; ProteinTech Group), and HRP-conjugated AffiniPure Goat Anti-Mouse IgG (H + L) (1:5000; Protein-Tech Group). The proteins were visualized using a chemiluminescence imaging analysis system (ECL; Tanon 5200; Shanghai Tianneng Technology Co., Shanghai, China), and band intensity was analyzed with ImageJ.

### Statistical Analysis

Data were statistically analyzed using the SPSS software (IBM, Chicago, IL, USA). Results are expressed as mean ± standard deviation and are representative of at least three independent experiments. Comparisons between the two groups were performed using independent sample *t-*tests, and differences among multiple groups were compared with one-way analysis of variance. The results were considered significant for *P* < 0.05.

## Results

### SIRT1 Expression Decreased with Subculturing

**Fig. 1:**
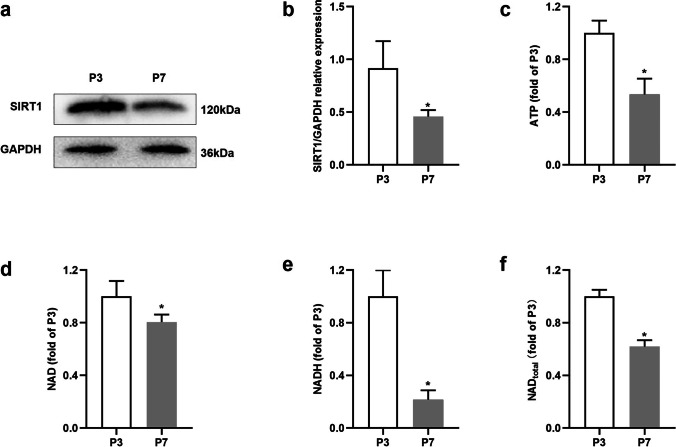
SIRT1 expression decreases with subculture. (**a**, **b**) Western blotting results showing the protein expression levels of SIRT1 in HF-MSCs at P3 and P7. (**c**) The ATP levels of HF-MSCs at P3 and P7. (**d**, **e**, **f**) The levels of NAD, NADH, total NAD in HF-MSCs at P3 and P7. (**a**—**f**) *Compared with P 3, *P* < 0.05

SIRT1 protein and transcriptional expression levels decrease in mammalian tissues such as liver, kidney, and brain as a function of age [22–24]. As expected, Western blotting results showed that the expression of SIRT1 in HF-MSCs decreased with subculturing (*P* < 0.05; Fig. 1a, b). Moreover, the levels of total NAD, NADH, NAD, and ATP in HF-MSCs also decreased with subculturing (*P* < 0.05; Fig. 1c–f), with P7 (seventh-generation passage cells) showing 0.62, 0.22, 0.81, and 0.53 times lower levels than those observed in P3 (third-generation passage cells), respectively (*P* < 0.05; Fig. 1c–f).

### SIRT1 Knockdown Enhanced Cellular Senescence and Apoptosis, Accompanied by Increased DNA Damage Aggravation and NAD as well as ATP Depletion

**Fig. 2:**
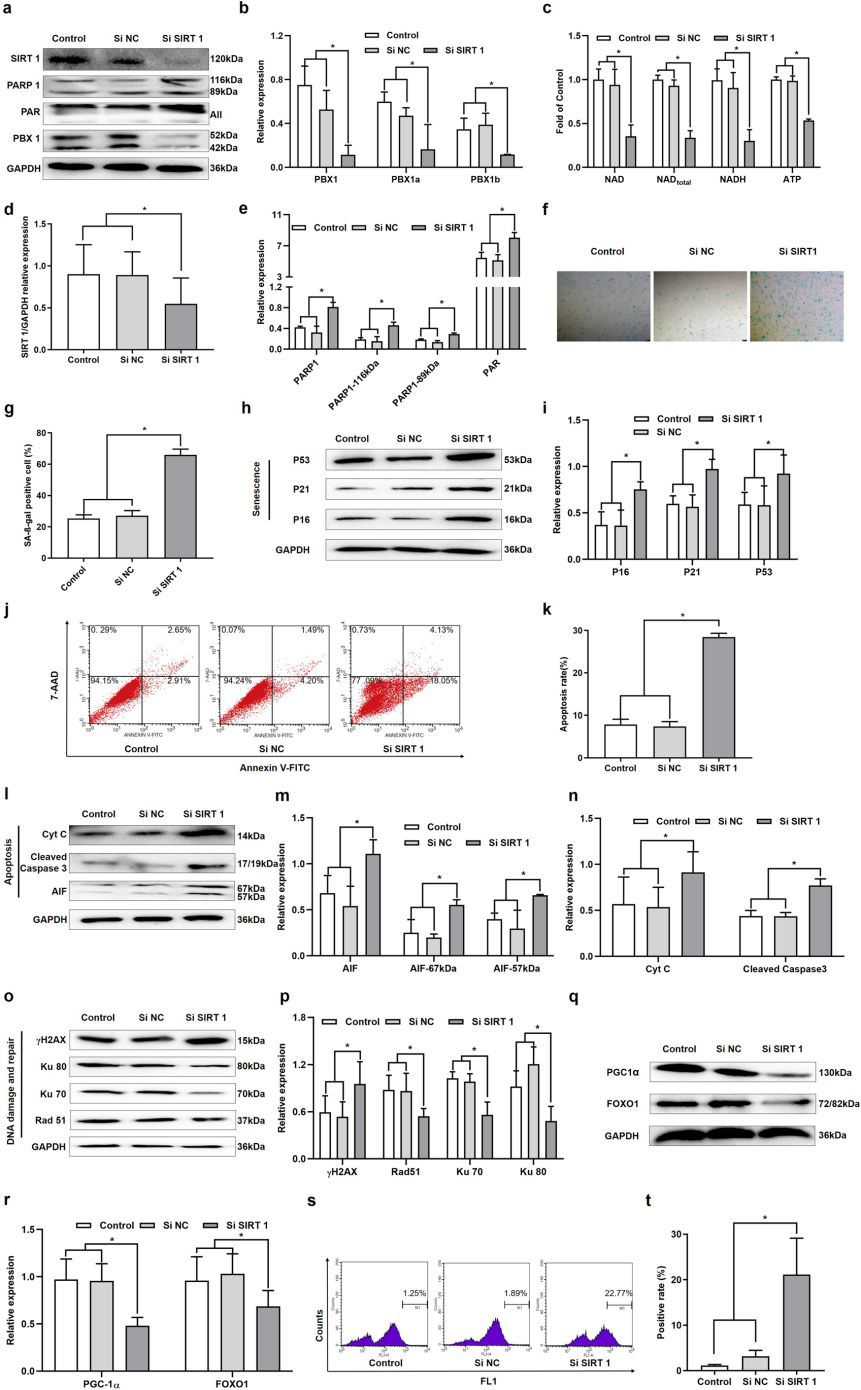
SIRT1 knockdown enhances cellular senescence and apoptosis, which is accompanied by increased DNA damage aggravation and NAD and ATP depletion. (**a**) Western blotting analysis of the protein expression levels of PBX1, SIRT1, PAR and PARP1 after SIRT1 knockdown in HF-MSCs. (**b**) The relative expression levels of PBX1 in HF-MSCs after SIRT1 knockdown in HF-MSCs. (**c**) Levels of ATP, NAD, NADH and total NAD in HF-MSCs after SIRT1 knockdown. (**d**, **e**) The relative expression levels of SIRT1, PAR and PARP1 in HF-MSCs after SIRT1 knockdown in HF-MSCs. (**f**, **g**) HF-MSC SA-β-gal staining results after SIRT1 knockdown (Scale bar = 200 μm). (**h**, **i**) Western blotting analysis of the protein expression levels of P53, P21 and P16 after SIRT1 knockdown in HF-MSCs. (**j**, **k**) Flow cytometry results of HF-MSCs apoptosis after SIRT1 knockdown. (**l**, **m**, **n**, **o**, **p**, **q**, **r**) Western blotting analysis of the protein expression levels of AIF, cleaved caspase 3, Cyt C, Ku70, Ku80, Rad 51, γH2AX PGC1α and FOXO1 after SIRT1 knockdown in HF-MSCs. (**s**, **t**) Flow cytometry results of the ROS levels in HF-MSCs after SIRT1 knockdown. (**a**—**t**) *P < 0.05

HF-MSCs entered replicative senescence and apoptosis [21], which was accompanied by downregulation of SIRT1 expression. To further confirm whether the inhibition of SIRT1 promotes cellular senescence and apoptosis, we knocked down SIRT1 in HF-MSCs. SIRT1 knockdown decreased the expression of PBX1 (*P* < 0.05; Fig. 2a, b) and reduced the levels of intracellular total NAD (*P* < 0.05; Fig. 2c), NADH (*P* < 0.05; Fig. 2c), NAD (*P* < 0.05; Fig. 2c), and ATP (*P* < 0.05; Fig. 2c) compared to those observed in the SiNC group. Moreover, SIRT1 knockdown increased PARP1 expression compared to those observed in the SiNC group (*P* < 0.05; Fig. 2a, e).

As expected, western blotting results showed that SIRT1 knockdown upregulated the expression of 1) senescence-related proteins such as P53, P21, and P16 (*P* < 0.05; Fig. 2h, i); 2) apoptosis-related proteins such as cleaved caspase 3, Cyt C, and 57-kDa AIF (*P* < 0.05; Fig. 2l, m, and n); and 3) DNA damage-related protein γH2AX (*P* < 0.05; Fig. 2o, p) compared to those observed in the SiNC group. Thus, the western blotting results of senescence and apoptosis-related proteins were in agreement with the results of 7-AAD Annexin V-FITC (*P* < 0.05; Fig. 2j, k) and SA-β-gal (*P* < 0.05; Fig. 2f, g). The percentage of SA-β-gal-positive (Fig. 2f, g), 7-AAD Annexin V-FITC-positive (Fig. 2j, k), and ROS-positive (Fig. 2s, t) cells in the SiSIRT1 group was increased compared to the SiNC group. Furthermore, SIRT1 knockdown inhibited the expression of PGC1α and FOXO1 (*P* < 0.05; Fig. 2q, r) compared to those observed in the SiNC group.

### PBX1 Rescued ROS-Mediated HF-MSCs Senescence and Apoptosis, Accompanied by Decreased DNA Damage Aggravation

Human cells are often exposed to and damaged by various endogenous and exogenous damage factors, accompanied by production of ROS which is one of the common products of cell damage. Excessive ROS accumulation is regarded as major risk factors for cellular damage which includes but is not limited to cellular senescence and apoptosis. In our study, HF-MSCs were treated with H_2_O_2_ at the concentration of 0, 50, 75, 100, and 125 μM for 2 h to mimic the effects of ROS on HF-MSCs. The percentage of SA-β-gal-positive cells and Annexin V-PI-FITC-positive cells in HF-MSCs at 100 μM and 125 μM group increased than that at control group (*P* < 0.05; Figures S1a, b, e, f). In agreement with the results of the senescence and apoptosis assays, the westernblot assay demonstrated that the expression levels of the senescence-associated proteins P16, P53, and P21 (*P* < 0.05; Figures S1c, d), and apoptosis-associated proteins cleaved caspase 3 (*P* < 0.05; Figures S1h, i) were increased at 100 and 125 μM group than that at control group. DNA damage aggravation with increased the concentration of H_2_O_2_ was observed in HF-MSCs, as shown by the increased expression of γH2AX in HF-MSCs at 100 μM and 125 μM compared to control (*P* < 0.05; Figures S1j, k). In contrast, PBX1 overexpression in H_2_O_2_-treated and -untreated HF-MSCs significantly decreased the percentage of apoptotic positive cells (*P* < 0.05; Figures S2c, d), and that of SA-β-gal-positive cells (*P* < 0.05; Figures S2a, Sb). PBX1 overexpression in H_2_O_2_-treated and -untreated HF-MSCs significantly reduced the expression of DNA damage-related proteins γH2AX (*P* < 0.05; Figures S2e, f).

### PBX1 Rescued SIRT1 Knockdown-Mediated HF-MSCs Senescence and Apoptosis by Alleviating ROS-Mediated DNA Damage and Intracellular NAD Depletion

**Fig. 3:**
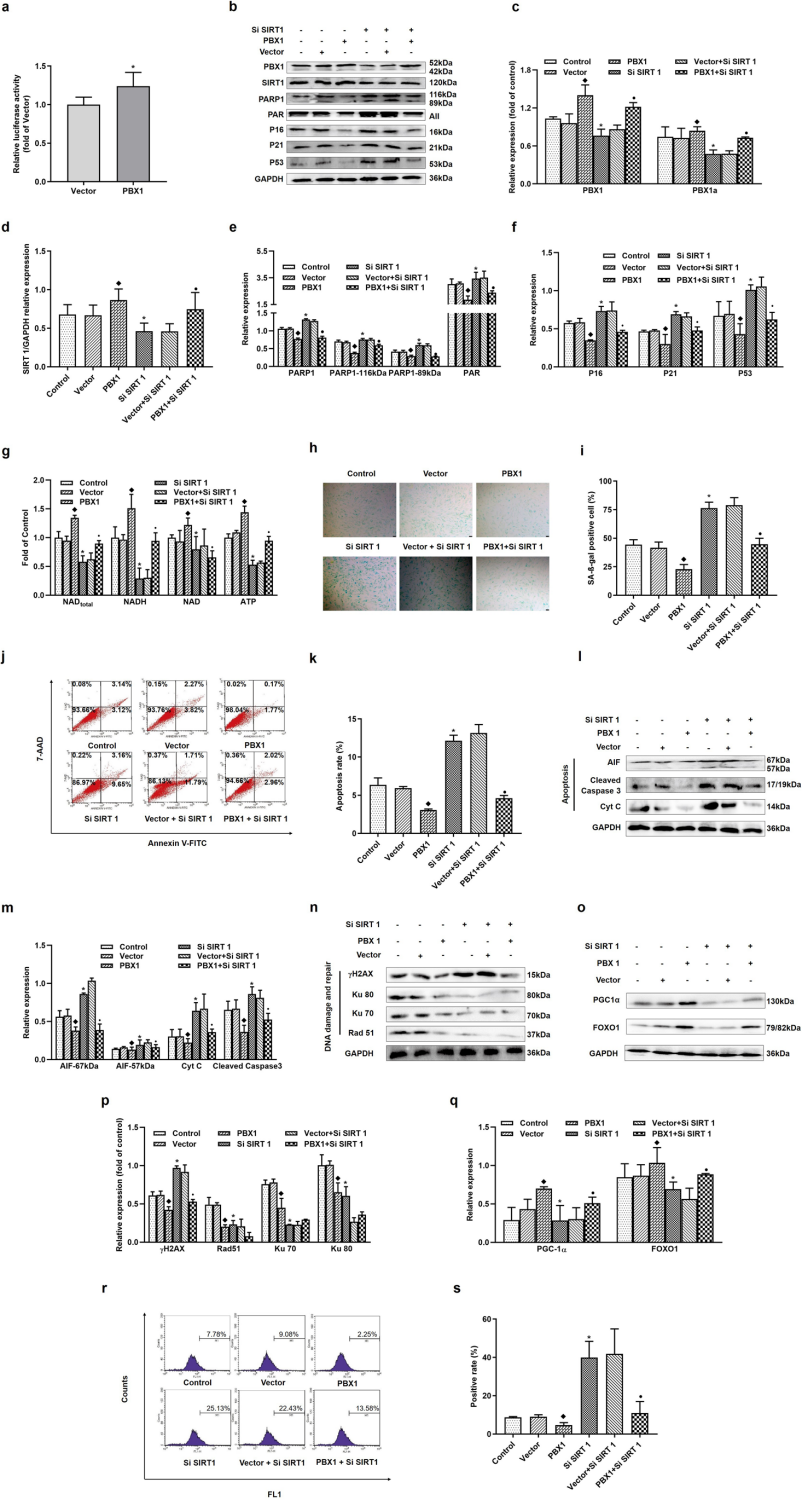
PBX1 rescues SIRT1 knockdown-mediated HF-MSC senescence and apoptosis by alleviating ROS-mediated DNA damage and intracellular NAD depletion. (**a**) Dual-luciferase reporter gene results of the luciferase activity of the SIRT1 promoter after PBX1 overexpression. (**b**, **c**, **d**, **e**, **f**) Western blotting analysis of the protein expression levels of PBX1, P53, P21, P16, SIRT1, PAR and PARP1 after PBX1 overexpression in the SIRT1 knockdown group. (**g**) The level of ATP, NAD, NADH, and total NAD in HF-MSCs after PBX1 overexpression in the SIRT1 knockdown group. (**h**, **i**) HF-MSCs SA-β-gal staining results after PBX1 overexpression in the SIRT1 knockdown group (Scale bar = 200 μm). (**j**, **k**) Flow cytometry results of HF-MSCs apoptosis after PBX1 overexpression in the SIRT1 knockdown group. (**l**, **m**, **n**, **o**, **p**, **q**) Western blotting analysis of the protein expression levels of AIF, cleaved caspase 3, Cyt C, Ku70, Ku80, Rad 51, γH2AX, PGC1α and FOXO1 after PBX1 overexpression in the SIRT1 knockdown group. (**r**, **s**) Flow cytometry results of ROS levels in HF-MSCs after PBX1 overexpression in the SIRT1 knockdown group. (**a**—**s**) *Compared with Control, ♦comapred with Vector, ●comapred with Vector + Si SIRT 1, *P* < 0.05

As described, SIRT1 positively regulates cell survival. Therefore, SIRT1 regulation might represent a plausible strategy for the treatment of aging and aging-related diseases. Our previous studies have shown that PBX1 promotes HF-MSCs proliferation and attenuates cellular senescence as well as apoptosis [21]. To further confirm whether PBX1 could attenuate HF-MSCs senescence and apoptosis by upregulating SIRT1 and whether PBX1 could rescue SIRT1-knockdown-mediated HF-MSCs senescence and apoptosis, we overexpressed PBX1 and knocked down SIRT1 in HF-MSCs. A dual-luciferase reporter gene assay showed that the luciferase activity associated with the SIRT1 promoter in the PBX1 group was significantly higher than that in the vector group (*P* < 0.05; Fig. 3a). Furthermore, we discovered that compared with the control group, the SIRT1 knockdown group showed a significant increase in the percentage of apoptotic (*P* < 0.05; Fig. 3j, k), senescent (*P* < 0.05; Fig. 3h, i), and ROS-positive cells (*P* < 0.05; Fig. 3r, s). In contrast, the PBX1-overexpressing group showed a decrease in the percentages of apoptotic (*P* < 0.05; Fig. 3j, k), senescent (*P* < 0.05; Fig. 3h, i), and ROS-positive cells (*P* < 0.05; Fig. 3r, s) compared to those observed in the empty vector group. Furthermore, the percentage of SA-β-gal-positive cells (*P* < 0.05; Fig. 3h, i), 7-AAD Annexin V-FITC-positive cells (*P* < 0.05; Fig. 3j, k), and ROS-positive cells (*P* < 0.05; Fig. 3r, s) in the PBX1 + SiSIRT1 group was decreased compared to the vector + SiSIRT1 group.

Western blotting results showed that SIRT1 knockdown significantly upregulated the expression of P53, P21, and P16 (*P* < 0.05; Fig. 3b, f); 57-kDa AIFs, cleaved caspase 3, and Cyt C (*P* < 0.05; Fig. 3l, m); and γH2AX (*P* < 0.05; Fig. 3n, p) compared to those observed in the control group. Moreover, PBX1 overexpression significantly downregulated the expression levels of P53, P21, and P16 (*P* < 0.05; Fig. 3b, f) and 57-kDa AIF, cleaved caspase 3, and Cyt C (*P* < 0.05; Fig. 3l, m) compared to the effect of the empty vector. Further, PBX1 + SiSIRT1 downregulated the expression of P53, P21, and P16 (*P* < 0.05; Fig. 3b, f); 57-kDa AIF, cleaved caspase 3, and Cyt C (*P* < 0.05; Fig. 3l, m); and γH2AX (*P* < 0.05; Fig. 3n, p) compared to the protein expression levels following vector + SiSIRT1. The percentage of ROS-positive cells was also decreased (*P* < 0.05; Fig. 3r, s). PBX1 + SiSIRT1 downregulated the expression of PARP1 and PAR (*P* < 0.05; Fig. 3b, e), upregulated the expression of SIRT1, PGC1α, and FOXO1 (*P* < 0.05; Fig. 3o, q), and increased the levels of intracellular total NAD, NADH, and ATP (*P* < 0.05; Fig. 3g) in comparison to those observed under the vector + SiSIRT1 condition. These data suggested that PBX1 rescues SIRT1-knockdown-mediated HF-MSCs senescence and apoptosis by alleviating ROS-mediated DNA damage and intracellular NAD depletion and that the SIRT1–PARP1 axis plays a critical role in PBX1-alleviated HF-MSCs senescence and apoptosis.

### PBX1 Rescued PARP1 Overexpression-Mediated ATP and NAD Depletion, Accompanied by Increased SIRT1 Expression

**Fig. 4:**
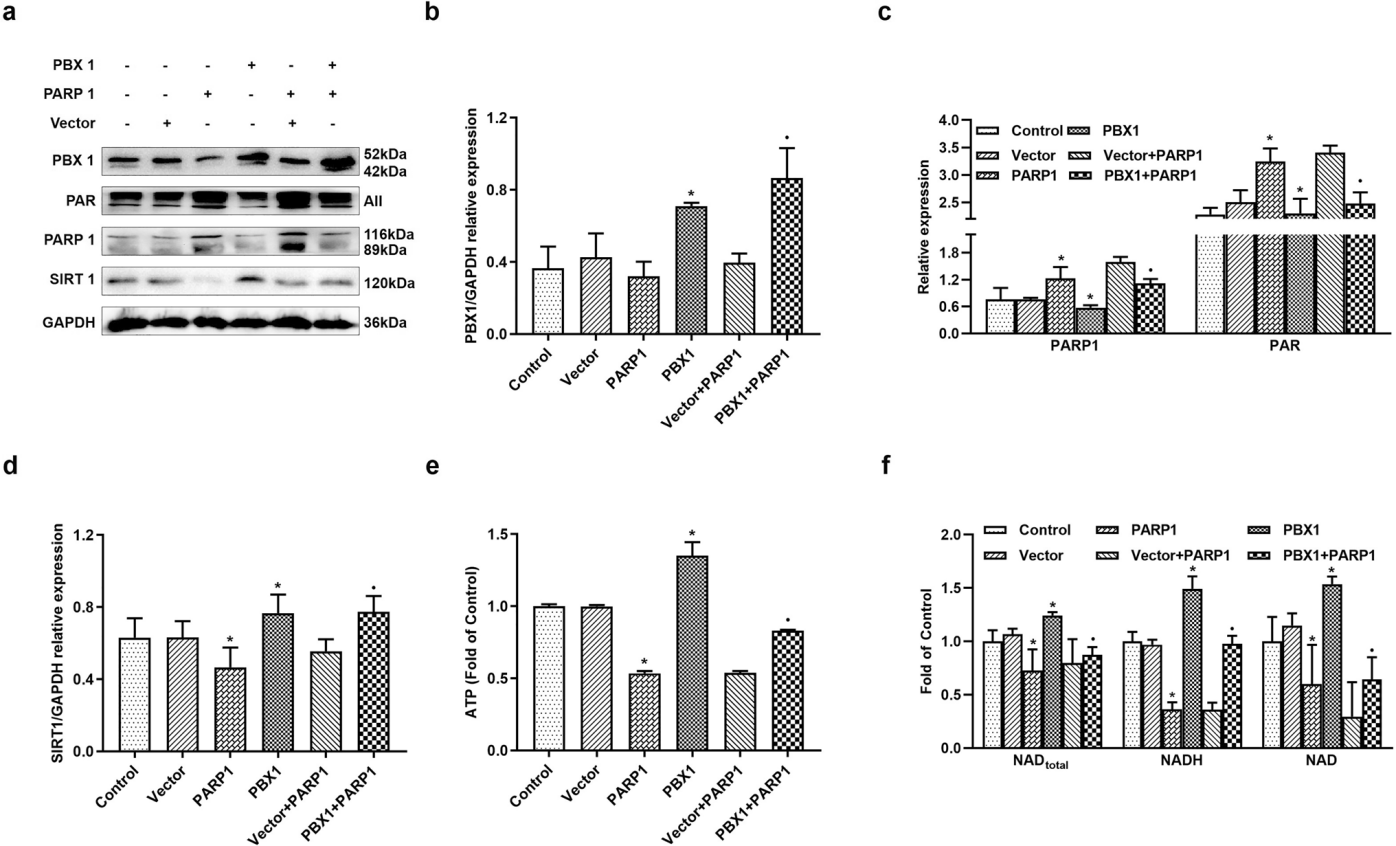
PBX1 rescued PARP1 overexpression-mediated HF-MSCs senescence and apoptosis, accompanied by increased SIRT1 expression and intracellular NAD and ATP levels. (**a**, **b**, **c**, **d**) Western blotting analysis of the protein expression levels of PBX1, SIRT1, PAR and PARP1 after PBX1 overexpression in PARP1-overexpressing cells. (**e**, **f**) Levels of ATP, NAD, NADH, total NAD in HF-MSCs after PBX1 overexpression in PARP1-overexpressing cells. (**a**—**f**) *Compared with Vector, ●comapred with Vector + RARP1, *P* < 0.05

We demonstrated that PBX1 rescued SIRT1 knockdown-mediated HF-MSCs senescence and apoptosis, accompanied by decreased PARP1 expression. In contrast, SIRT1 knockdown significantly upregulated the expression levels of PARP1. Since NAD is a major shared substrate between SIRT1 and PARP1, we generated PARP1-, PBX1-, and PARP1 + PBX1-overexpressing HF-MSCs to ascertain whether the PARP1–SIRT1 axis plays a role in PBX1-mediated alleviation of cellular senescence and apoptosis. Western blotting results showed that both PARP1 and PBX1 overexpression increased SIRT1 levels (*P* < 0.05; Fig. 4a, d) compared to those observed following PARP1 vector overexpression.

Our results showed that the PARP1-overexpressing group exhibited a significantly decreased SIRT1 expression (*P* < 0.05; Fig. 4a, d) as well as total NAD (*P* < 0.05; Fig. 4f), NADH (*P* < 0.05; Fig. 4f), and ATP (*P* < 0.05; Fig. 4e) levels compared to those observed in the empty vector group. In contrast, the PBX1-overexpressing group increased the expression of SIRT1 (*P* < 0.05; Fig. 4a, d) as well as total NAD (*P* < 0.05; Fig. 4f), NADH (*P* < 0.05; Fig. 4f), and ATP (*P* < 0.05; Fig. 4e) levels compared to those observed in the empty vector group. Furthermore, PARP1 + PBX1 overexpression increased the expression of SIRT1 (*P* < 0.05; Fig. 4a, d) and total NAD (*P* < 0.05; Fig. 4f), NADH (*P* < 0.05; Fig. 4f), and ATP (*P* < 0.05; Fig. 4e) levels compared to those observed following vector + PARP1 overexpression. Surprisingly, compared to the empty vector group, the PBX1-overexpressing group downregulated PARP1 expression (*P* < 0.05; Fig. 4a, c) and upregulated SIRT1 expression (*P* < 0.05; Fig. 4a, d); thus, suggesting that the PARP1–SIRT1 axis plays a role in the PBX1-mediated alleviation of cellular senescence and apoptosis and that PBX1 rescued PARP1 overexpression-mediated ATP and NAD depletion, accompanied by increased SIRT1 expression.

## Discussion

In this study, we demonstrated that PBX1 alleviates HF-MSCs senescence and apoptosis by reducing ROS-mediated DNA damage through the regulation of the SIRT1–PARP1 axis. In this study, subculturing HF-MSCs decreased SIRT1 expression and increased ATP as well as intracellular NAD depletion. This was consistent with the observation that SIRT1 expression diminishes with age in mammals. The SIRT1 degradation, which is commonly considered an autophagy substrate by the autophagosome-lysosome, contributes to the loss of SIRT1 during cellular senescence [18]. SIRT1 is regarded as a crucial epigenetic regulator that facilitates DNA repair and genome stability preservation by depleting a large amount of NAD. The SIRT1 levels, which represent a major source of cellular NAD consumption, can directly affect the activity of other NAD-dependent enzymes, such as PARP1. SIRT1 is widely involved in the anti-aging process in various organisms, including yeast, worms, and mammals [7]. Our previous studies demonstrated that HF-MSCs enter replicative senescence and apoptosis, which is accompanied by decreased PARP1 expression[21]. Accumulating evidence suggests that diminished NAD levels during aging result in mitochondrial and stem cell dysfunction as well as accumulation of DNA damage via the induction of DBC1 binding to PARP1 and SIRT1 and their subsequent inhibition [25]. Therefore, various novel strategies to regulate the SIRT1–PARP1 axis either directly or indirectly may demonstrate therapeutic potential in attenuating senescence and apoptosis, which are known to be involved in aging and aging-related diseases.

Furthermore, SIRT1 catalyzes the deacetylation of the acetyl-lysine residues of histone proteins H1, H3, and H4. Additionally, SIRT1 deacetylates non-histone substrates, including P53, Ku70, FoxOs, PGC1-a, PPAR-γ, AMPK, mTOR, MyoD, and NF-κB [26, 27], which are all related to delayed cellular senescence. SIRT1 is widely involved in the regulation of cellular senescence and organismal longevity via the acetylation and deacetylation of these substrates, and by altering their transcriptional and enzymatic activities as well as protein levels [7, 10]. Thus, SIRT1 upregulation can affect the regulation of cellular senescence in aging and aging-related diseases. To further confirm whether the inhibition of SIRT1 promotes cellular senescence and apoptosis, we knocked down SIRT1 in HF-MSCs. As expected, the inhibition of SIRT1 led to intracellular NAD depletion and increased PARP1 activity accompanied by increased DNA damage aggravation, cellular senescence, and apoptosis, which were correlated with the increased expression of proteins associated with cellular senescence (P53, P16, and P21), apoptosis (cleaved caspase 3, and Cyt C), and DNA damage (γH2AX). SIRT1 deletion in hematopoietic stem cells (HSCs) induces characteristic changes normally associated with HSC senescence, such as genomic instability and increased sensitivity to DNA damage [28]. Our data also showed that SIRT1 inhibition led to a decrease in FOXO1 and PGC1α expression, which implies that SIRT1 knockdown could reduce the ability against oxidative injury.

The pivotal function of SIRT1 is displayed through specific interactions with P53, namely P53 deacetylation at the C-terminal lysine-382 residue in a NAD-dependent manner [29]. This interaction reduces P53-mediated transcriptional and translational levels as well as the expression of its downstream proteins, including the cyclin-dependent kinase inhibitors P21 and P16, which have been shown to participate in the regulation of cell cycle arrest, cellular senescence, and apoptosis [29]. Therefore, SIRT1 can inhibit P53-dependent cell cycle arrest, cellular senescence, and apoptosis while also activating the genes involved in DNA damage repair to promote cell survival and cell proliferation. This implies that regulation of the SIRT1–P53 axis can regulate stem cell fate, which influences tissue homeostasis, regeneration, and repair. In our study, SIRT1 knockdown decreased PBX1 expression, and a previous study showed that SIRT1 deficiency also reduces PBX1 binding to the apoptotic cell response element of the IL-10 promoter [30]. Conversely, the activation of SIRT1 ultimately activates PBX1 [30]. PBX1 is a key transcription factor that participates in the regulation of stem cell fate by cooperating with Oct 4, Nanog, and Sox2 [31, 32], while also participating in stem cell proliferation, apoptosis, senescence, and differentiation to maintain stem cells in a pluripotent and undifferentiated state [13–15]. Our recent studies showed that PBX1 enhances HF-MSCs proliferation and their reprogramming into induced pluripotent stem cells, and attenuates their senescence [15]. Here, we showed that increased expression of PBX1 attenuates HF-MSCs senescence and apoptosis; thus, suggesting that PBX1 may be involved in HF-MSCs senescence, which is consistent with the findings of our previous study [21].

To further confirm whether PBX1 could attenuate HF-MSCs senescence and apoptosis by upregulating SIRT1, and whether PBX1 could rescue SIRT1 knockdown-mediated HF-MSCs senescence and apoptosis, we overexpressed PBX1 and knocked down SIRT1 in HF-MSCs. The results showed that the increased expression of PBX1 rescued HF-MSCs senescence and apoptosis caused by SIRT1 knockdown. Increased PBX1 expression enhanced the luciferase activity of the SIRT1 promoter in a dual-luciferase reporter gene assay, which indicates that PBX1 promotes SIRT1 transcription by binding to the SIRT1 promoter. Furthermore, increased PBX1 expression attenuated ROS accumulation, DNA damage aggravation, and intracellular NAD depletion, regardless of whether SIRT1 was knocked down. Thus, PBX1 rescued SIRT1 knockdown-mediated HF-MSCs senescence and apoptosis by alleviating ROS-mediated DNA damage and intracellular NAD depletion.

Both SIRT1 and PARP1 utilize NAD as a substrate. PARP1 uses poly(ADP-ribose)s (PARs) converted from NAD, and SIRT1 uses NAD as its cofactor [33]. Considering that SIRT1 is the main consumer of cellular NAD, the SIRT1 level can directly affect the level and activation of other NAD-dependent enzymes, such as PARP1, which plays a crucial role in the protection against oxidative injury. PARP1 has dual effects, depending on the cellular environment. PARP1 is generally regarded as a DNA damage repair enzyme that exerts a protective effect by recruiting repair-related proteins to sites with minor DNA damage to facilitate DNA damage repair [34]. In contrast, PARP1 overactivation due to depletion of large amounts of NAD and ATP, leads to an energy crisis and parthanatos, a regulated type of necrosis that occurs in response to extensive DNA damage [35]. Upon PARP1 activation, the polyADP-ribosylation process dissociates histone H1 from the FoxO3a target gene promoter and facilitates FoxO3a nuclear accumulation and binding to its target promoters, resulting in improved expression of autophagy-related genes. PARP1-induced autophagy activation impairs mitochondrial metabolism and accelerates cellular senescence and death [36]. Our previous study showed that PARP1 overexpression significantly aggravated HF-MSCs senescence and apoptosis[21] and decreased the expression of SIRT1; thus, suggesting that regulating the SIRT1-PARP1 axis would be advantageous for attenuating senescence and apoptosis involved in aging and aging-related diseases. To explore whether PBX1 participates in the attenuation of cellular senescence and apoptosis in HF-MSCs, and elucidate the mechanism underlying its mode of action, we generated HF-MSCs overexpressing PBX1, PARP1, or both. As expected, increased PBX1 expression significantly reduced intracellular NAD and ATP depletion and also increased SIRT1 expression in HF-MSCs, suggesting that PBX1 participates in the attenuation of cellular senescence and apoptosis in HF-MSCs, possibly by interfering with the SIRT1-PARP1 axis. In contrast, PARP1 overexpression increased NAD and ATP depletion in HF-MSCs. Interestingly, compared to the PARP1 + vector overexpression condition, both PBX1 and PARP1 overexpression conditions were associated with reduced NAD and ATP depletion in HF-MSCs. Thus, our data showed that PBX1 overexpression downregulated PARP1 expression and upregulated SIRT1 expression, suggesting that SIRT1 inhibits PARP1 activity. This implies that the SIRT1-PARP1 axis plays a role in cellular senescence and apoptosis.

These data further demonstrate that the NAD-SIRT1-PARP1 axis plays a critical role in PBX1-alleviated HF-MSCs senescence and apoptosis. PBX1 alleviates HF-MSCs senescence and apoptosis by reducing ROS-mediated DNA damage via the SIRT1-PARP1 axis. This provides a new perspective on the underlying mechanism of stem cell senescence and lays the foundation for age-related disease prevention and treatment.

However, SIRT1 mediates positive regulatory influences on cell survival, whereas PARP1 kills or protects cells, depending on the severity of the insult. Therefore, manipulation of the SIRT1-PARP1 axis seems to represent a plausible strategy for the treatment of aging and age-related diseases. However, PARP1 inhibitory drugs on the market, which target the catalytic activity of PARP1, might affect diverse biochemical functions of PARP1 [37]. In contrast, drugs selectively affecting the SIRT1-PARP1 interaction would not exert unnecessary side effects on desirable functions mediated by PARP1. Therefore, the development of a specific regulator targeting the SIRT1–PARP1 axis would be advantageous for the treatment of aging and aging-related diseases.

## Conclusion

**Fig. 5:**
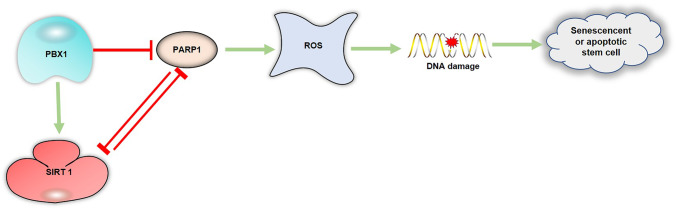
PBX1 alleviates HF-MSCs senescence and apoptosis by reducing oxidative stress-mediated DNA damage via interaction with the PARP1-SIRT1 axis

In this study, we demonstrated that PBX1 attenuates intrinsic ROS-mediated HF-MSCs senescence and apoptosis by regulating the SIRT1–PARP1 axis (Fig. 5). Meanwhile our results revealed that a positive interaction feedback loop exists between PBX1 and SIRT1. Furthermore, we provide a foundation for the development of a specific regulator targeting the SIRT1–PARP1 axis, which would be advantageous for the prevention and treatment of aging and aging-related diseases, especially for hair follicle repair and regeneration.

## Supplementary Information

Below is the link to the electronic supplementary material.

Supplementary file1 Figure S 1. H_2_O_2_ treatment enhances cellular senescence and apoptosis, which is accompanied by increased DNA damage aggravation. (a, b) The SA-β-gal staining results after H_2_O_2_ treatment (Scale bar = 200 μm). (c, d) Western blotting analysis of the protein expression levels of P53, P21 and P16 after H_2_O_2_ treatment in HF-MSCs. (e, f) Flow cytometry results of HF-MSCs apoptosis after H_2_O_2_ treatment. (g) Annexin V-PI staining results of HF-MSCs apoptosis after H_2_O_2_ treatment. (h, i) Western blotting analysis of the protein expression levels of AIF, cleaved caspase 3 and Cyt C after H_2_O_2_ treatment in HF-MSCs. (j, k, l) Western blotting analysis of the protein expression levels of Ku70, Ku80, Rad 51, γH2AX, PARP 1 and PAR after H_2_O_2_ treatment in HF-MSCs. (a—l) *Compared with Control, *P* < 0.05 (JPG 1333 KB)

Supplementary file2 Figure S 2. PBX1 rescued ROS-mediated HF-MSCs senescence and apoptosis, accompanied by decreased DNA damage aggravation. (a, b) The SA-β-gal staining results of H_2_O_2_-treated HF-MSCs after PBX1 overexpression. (Scale bar = 200 μm). (c, d) Flow cytometry results of HF-MSCs apoptosis of H_2_O_2_-treated HF-MSCs after PBX1 overexpression. (e, f) Western blotting analysis of the protein expression levels of γH2AX in H_2_O_2_-treated HF-MSCs after PBX1 overexpression. (a—f) *Compared with Vector, ●comapred with Control, ■compared with Vector + H_2_O_2_, *P* < 0.05 (JPG 636 KB)

## Data Availability

The data underlying this article will be shared on reasonable request to the corresponding author.

## Abbreviations

DDR: DNA damage response
HDACs: Histone deacetylases
HATs: Histone acetylases
HFs: Hair follicles
HF-MSCs: Hair follicle-derived mesenchymal stem cell
NAD: Nicotinamide adenine dinucleotide
NNM: Nicotinamide mononucleotide
NR: Nicotinamide riboside
HSCs: Hematopoietic stem cells
P3: Third-generation passage cells
P7: Seventh-generation passage cells
PARs: Poly(ADP-ribose)s
PARP 1: Poly (ADP-ribose) polymerase-1
PBX1: Pre-B-cell leukemia transcription factor1
ROS: Reactive oxyden species
SIRT 1: Silent Information Regulator 1
