# Supplementary figures and images for: PBX1-SIRT1 Positive Feedback Loop Attenuates ROS-Mediated HF-MSC Senescence and Apoptosis
Source: Stem Cell Rev Rep. 2022 Aug 12;19(2):443–54. doi: 10.1007/s12015-022-10425-w (PMC9902417; doi:10.1007/s12015-022-10425-w)

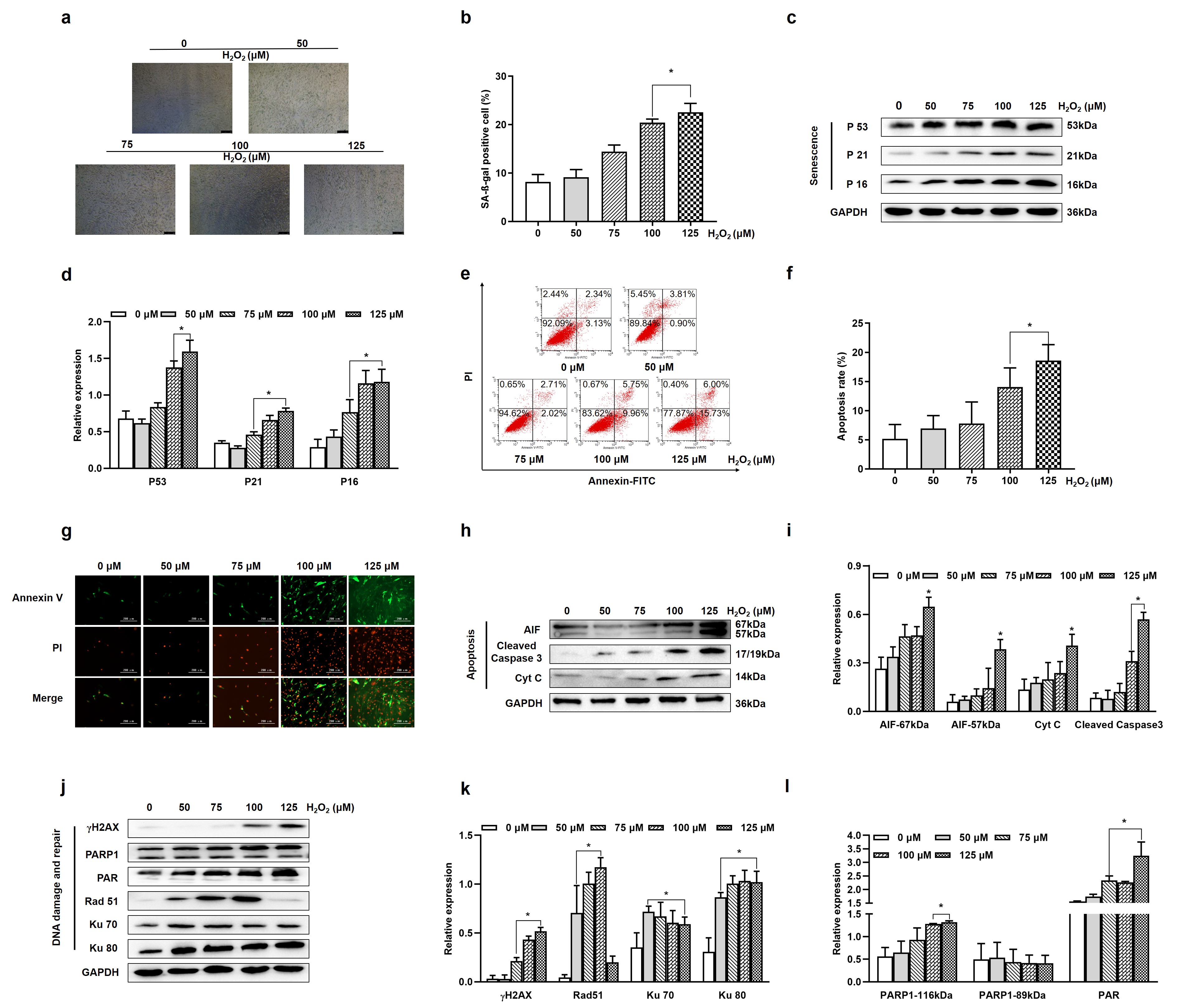

Supplement: Supplementary file 1 — Supplementary file1 Figure S 1. H2O2 treatment enhances cellular senescence and apoptosis, which is accompanied by increased DNA damage aggravation. (a, b) The SA-β-gal staining results after H2O2 treatment (Scale bar = 200 μm). (c, d) Western blotting analysis of the protein expression levels of P53, P21 and P16 after H2O2 treatment in HF-MSCs. (e, f) Flow cytometry results of HF-MSCs apoptosis after H2O2 treatment. (g) Annexin V-PI staining results of HF-MSCs apoptosis after H2O2 treatment. (h, i) Western blotting analysis of the protein expression levels of AIF, cleaved caspase 3 and Cyt C after H2O2 treatment in HF-MSCs. (j, k, l) Western blotting analysis of the protein expression levels of Ku70, Ku80, Rad 51, γH2AX, PARP 1 and PAR after H2O2 treatment in HF-MSCs. (a—l) *Compared with Control, P < 0.05 (JPG 1333 KB) [file 12015_2022_10425_MOESM1_ESM.jpg]

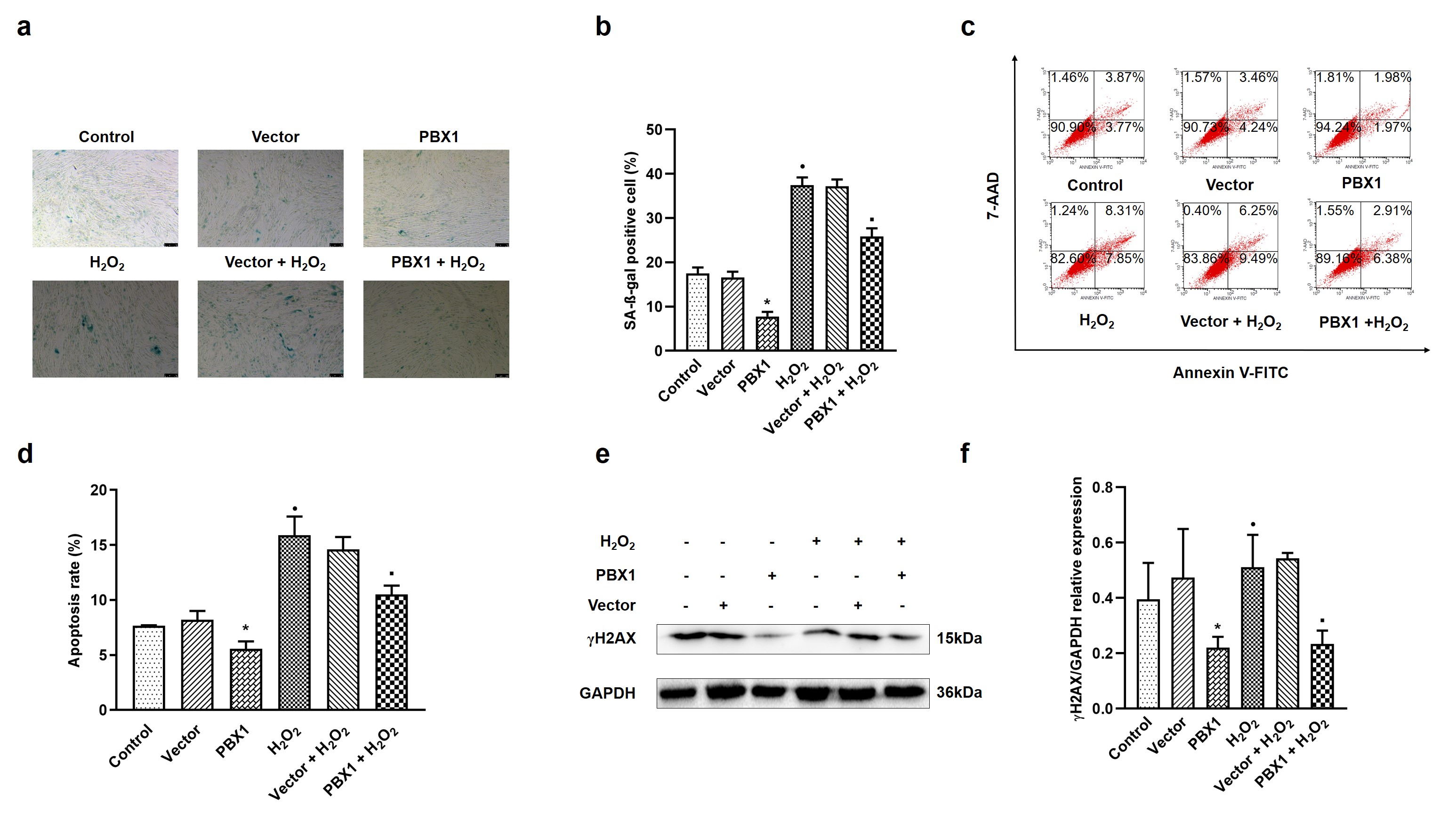

Supplement: Supplementary file 2 — Supplementary file2 Figure S 2. PBX1 rescued ROS-mediated HF-MSCs senescence and apoptosis, accompanied by decreased DNA damage aggravation. (a, b) The SA-β-gal staining results of H2O2-treated HF-MSCs after PBX1 overexpression. (Scale bar = 200 μm). (c, d) Flow cytometry results of HF-MSCs apoptosis of H2O2-treated HF-MSCs after PBX1 overexpression. (e, f) Western blotting analysis of the protein expression levels of γH2AX in H2O2-treated HF-MSCs after PBX1 overexpression. (a—f) *Compared with Vector, ●comapred with Control, ■compared with Vector + H2O2, P < 0.05 (JPG 636 KB) [file 12015_2022_10425_MOESM2_ESM.jpg]
